# A Qualitative Study with Healthcare Staff Exploring the Facilitators and Barriers to Engaging in a Self-Help Mindfulness-Based Intervention

**DOI:** 10.1007/s12671-017-0740-z

**Published:** 2017-05-25

**Authors:** Moitree Banerjee, Kate Cavanagh, Clara Strauss

**Affiliations:** 1 School of Psychology, University of Sussex, Brighton, BN1 9QH UK; 2 Sussex Partnership NHS Foundation Trust, Sussex Education Centre, Mill View Hospital, Nevill Avenue, Hove, BN3 7HY UK

**Keywords:** Engagement, Attrition, Dropout, Self-help, Self-guided, Thematic analysis, Mindfulness, MBCT, MBSR

## Abstract

In order to increase the cost-efficiency, availability and ease of accessing and delivering mindfulness-based interventions (MBIs), clinical and research interest in mindfulness-based self-help (MBSH) interventions has increased in recent years. Several studies have shown promising results of effectiveness of MBSH. However, like all self-help interventions, dropout rates and disengagement from MBSH are high. The current study explored the facilitators and barriers of engaging in a MBSH intervention. Semi-structured interviews with members of healthcare staff who took part in an MBSH intervention (*n* = 16) were conducted. A thematic analysis approach was used to derive central themes around engagement from the interviews. Analyses resulted in four overarching themes characterising facilitation and hindrance to engagement in MBSH. These are “attitude towards engagement”, “intervention characteristics”, “process of change” and “perceived consequences”. Long practices, emerging negative thoughts and becoming self-critical were identified as the key hindrances, whilst need for stress reduction techniques, shorter practices and increased sense of agency over thoughts were identified as the key facilitators. Clinical and research implications are discussed.

## Introduction

Mindfulness can be defined as a process of purposefully cultivating non-judgemental attention to experiences in the present moment (Kabat-Zinn 2003), leading to “nondual co-emergent awareness at the subtlest level of consciousness, free from all conceptual constructs and frames” (Kang and Whittingham 2010, p. 163). Mindfulness-based interventions (MBIs) aim to improve psychological health by enhancing trait mindfulness (Quaglia et al. 2016). Mindfulness-based stress reduction (MBSR) and mindfulness-based cognitive therapy (MBCT) are the two most widely available and well-researched MBIs (Khoury et al. 2013). There is evidence from meta-analyses of randomised controlled trials (RCTs) showing that MBIs can be effective at reducing the relative risk of depressive relapse in recurrent depression in full or partial remission (Kuyken et al. 2016), lowering depressive symptom severity in currently depressed individuals at post-intervention in comparison to control conditions (Strauss et al. 2014) and reducing stress in non-clinical populations in comparison to control conditions (Chiesa and Serretti 2009).

Despite this evidence for the effectiveness of MBIs, engaging in MBIs can prove challenging. A recent meta-analysis reported a median of 15.5% of dropout from MBIs ranging from 8% in one study to as high as 37% in another among people diagnosed with a current episode of an anxiety or depressive disorder (Strauss et al. 2014). Another meta-analysis of MBIs in non-clinical populations reported a dropout rate of 16.99%, ranging from 3% in one study to as high as 34.9% in another (Khoury et al. 2015). MBIs are intensive interventions typically recommending daily formal mindfulness practice as well as cultivating mindfulness in daily life activities (Kabat-Zinn 2003). Unsurprisingly, making time for daily mindfulness practice is commonly reported as a challenge (Wyatt et al. 2014). Increasing awareness of thoughts whilst not engaging with their content is often described as one of the most “uncomfortable experiences” (Wyatt et al. 2014, p. 223). The mind has a strong habitual tendency to wander to the content of thoughts, and the “detached observation” (Kabat-Zinn 1982, p. 34) of a constantly changing field is difficult to attain (Chambers et al. 2009). Direct engagement with negative thoughts during mindfulness practice can lead to an escalation of distress and a cycle of negative reinforcement (Bishop 2002). In addition, qualitative analyses on the experience of participating in MBIs have reported that participants can have difficulty in engaging in a mindfulness practice due to physical discomfort, self-doubt, a feeling of being trapped in the long practices and feeling exhausted or disoriented (Dobkin et al. 2012; Lomas et al. 2015).

In addition to barriers of engaging in MBIs, challenges of disseminating the 8-week group-based interventions may limit the reach of MBIs. Challenges of disseminating MBIs include lack of trained mindfulness teachers, cost of community groups (Boggs et al. 2014; Crane and Kuyken 2013), reticence to engage in group interventions and logistical challenges to fit courses in with work demands (Wyatt et al. 2014). Hence, in order to increase the cost-efficiency, availability and ease of accessing and delivering MBIs, research interest in mindfulness-based self-help (MBSH) interventions is growing. Self-help approaches might be more efficient in terms of costs and use of resource and are also acceptable “minimal interventions” for participants and therapists (Bower and Gilbody 2005, p. 11). Consequently, interest in MBSH has proliferated in the recent years and a variety of MBSH resources are now available such as self-help books, audio guides, online programmes and mindfulness smart phone apps (Cavanagh et al. 2014).

There is growing evidence of the effectiveness in MBSH interventions. Recent meta-analyses of randomised controlled trials (RCTs) have found that self-help mindfulness/acceptance-based interventions have significant benefits for mindfulness, depression and anxiety (Cavanagh et al. 2014; Spijkerman et al. 2016). Another quasi-experimental comparison study investigating the effect of web-based MBCT reported significant reductions in depressive severity and rumination and increased mindfulness in recurrently depressed participants in comparison to usual care (Dimidjian et al. 2014). In a non-clinical population, Cavanagh et al. (2013) reported reduced perceived stress and anxiety/depression symptoms after a 2-week MBSH program. Lever Taylor et al. (2014) conducted an RCT using the MBSH book “Mindfulness: a practical guide to finding peace in a frantic world” (Williams and Penman 2011). The results revealed significant improvements in anxiety, depression and stress scores in a student sample in comparison to the wait-list control condition. Hence, there is growing evidence that MBSH is associated with benefits to mental health and well-being in both clinical and non-clinical populations.

However, a common challenge in self-help-based psychological approaches is engagement, and rates of attrition from pure self-help interventions tend to be higher than supported interventions (Eysenbach 2005). A review reported that the average dropout rate in (non-mindfulness) self-help interventions is 31% (Melville et al. 2010). This is similar to average dropout rate from MBSH interventions (37%) reported in a recent meta-analysis (Cavanagh et al. 2014). However, these meta-analyses defined dropout as the percentage of participants completing post-intervention measures. It must be noted that engagement may or may not be related to completion of post-treatment measures. It may be possible for participants to engage in an intervention but not complete post-treatment measures, or vice versa. In nine studies that reported number of participants meeting the study-defined engagement criteria, more than half (52%) disengaged from the self-help interventions (Cavanagh et al. 2014). This may be higher than post-intervention measurement completion rates because completing post-treatment measures demands less involvement compared to engaging in an intervention (Holdsworth et al. 2014).

Despite the high rates of disengagement from MBSH, we know surprisingly little about reasons for engagement and disengagement and theory is poorly developed in the area. Therefore, a qualitative approach to understanding reasons for engagement and disengagement in MBSH is warranted to contribute to theory development. Qualitative studies on the experience of participating in group-based MBIs (i.e. not self-help) have explored the issues of engaging with the intervention. A recent meta-synthesis of 15 qualitative studies examined the experience of participating in guided mindfulness interventions for individuals with mental health difficulties (Wyatt et al. 2014). The main *struggles* identified include practical limitations such as finding time, difficulty grasping the core concepts of mindfulness, being overwhelmed by new concepts, low mood and feeling distressed as a result of practising mindfulness meditation due to increased awareness on difficult memories and feelings. However, it is unclear if these themes would apply to MBSH. One of the key differences in MBSH and group-based MBIs is the group process. The meta-synthesis reported that a major theme of the experience of participating in group-based MBIs was the *normalising and supportive process of the group* as one of the eight major analytic themes (Wyatt et al. 2014). Hence, across the 15 studies involving a total of 170 participants, the positive effect of the group was noted as one of the major contributors of the experience of engaging in MBIs. Another meta-ethnography of the experience of participating in face-to-face MBIs by individuals with physical and mental health problems identified an important role for *the group process* (Malpass et al. 2012). This synthesis pointed out that the group context had a normalising effect, reducing the sense of stigma felt by participants and overcoming the experience of isolation. It might be argued that the group context played a pivotal positive role in MBIs by enhancing a sense of “fellowship, camaraderie and connection” (Mackenzie et al. 2007, p. 64). The psychological processes therefore involved in engaging with MBSH, without a group context, may be different to that of face-to-face group-based MBIs, and this needs to be directly examined. This is particularly important given the substantially higher rates of disengagement with MBSH in comparison to face-to-face MBIs.

A recent thematic analysis of the experience of a group-based MBCT examined the factors that assisted and/or hindered engagement for patients with chronic pain (Moore and Martin 2015). They identified *belief in the programme*, *perception of control*, *struggles* and *acceptance of the presence of pain* as the key factors contributing to engagement. Participants with positive perception of effects of from the MBCT programme were most motivated to continue mindfulness practice. A feeling of being empowered and to be able to take control of one’s own behaviour and response facilitated engagement. The key struggles that the participants faced were inflated expectation that they would achieve pain control and time pressures. Lastly, accepting the pain without resistance facilitated engagement in the MBCT program. However, to the best of our knowledge, no studies have explored the facilitators and barriers of engagement to an MBSH intervention.

With the growing research and clinical interest in the accessible forms of MBSH, examining the facilitators and barriers of engagement is crucial in order to maximise engagement and thereby maximising opportunities to benefit. Our aim in this study was to identify facilitators and barriers to engagement in a non-guided MBSH intervention from participants’ narrative of the experience using thematic analysis. To the best of our knowledge, no qualitative study so far has focused on the factors of engagement in MBSH interventions.

## Method

This study was part of a larger feasibility study of MBSH with 31 healthcare staff. Quantitative findings from the feasibility study are currently being written up for publication. The first author of the current qualitative study was only involved in conducting the interviews and was not involved in the wider feasibility study. This was in order to provide a degree of independence from the wider study.

### Participants

The only inclusion criterion for the feasibility study was that staff members had to be in a clinical role in the participating mental health National Health Service (NHS) trust. All 31 participants from the feasibility study were given the opportunity to be interviewed, of whom 16 participants (52%) agreed to be interviewed. The participants age ranged from 24 to 60 with a mean of 43.81 years (sd = 10.29), and 15 (93.8%) were female. All the participants were white British.

### Procedure

All clinical staff in the participating mental health NHS trust were invited to take part in the feasibility study. The feasibility study was advertised to staff from the mental health NHS trust via advertisements posted on the trust intranet, posters displayed around workplaces, fliers distributed at events and emails. Written and oral information was provided to all participants. Participants had to complete the pre- and post-intervention questionnaire as a part of the feasibility study. Informed consent, including consent to record interviews, was obtained from all participants before the study commenced.

On receiving informed consent, participants were asked to contact the research team indicating their preference of the form of mindfulness-based self-help (MBSH) program—an MBSH book or access to an MBSH online program. Participants were free to select either an online or book-based MBSH course:

#### MBSH Book

The book “Mindfulness: a practical guide to finding peace in a frantic world” (Williams and Penman 2011) was the MBSH used in this study. The book is based on the 8-week MBCT course and teaches mindfulness practices and principles through text and a CD. Readers were advised to read one intervention chapter per week for the 8 weeks of the course. A recent RCT showed that this using this book had medium effect sizes on measures of stress, anxiety and depression in the student population in comparison to an inactive control condition (Lever Taylor et al. 2014). Out of the participants interviewed, nine had opted for the book-based intervention.

#### MBSH Online Program

The “BeMindful” (www.bemindful.com) website was used as the online version of the MBSH program. This course incorporates MBCT and MBSR elements and consists of eight interactive 30-min online sessions in addition to introductory and course-end videos. The class sequence is based on the MBCT course. A feasibility study reported that perceived stress, anxiety and depression reduced significantly at course completion and decreased further at 1-month follow-up, with effect sizes similar to face-to-face MBIs (Krusche et al. 2013). After completing the 8-week intervention, participants who agreed to take part in the qualitative study were contacted by the first author to schedule the telephone interview. Of the participants interviewed, seven participants had opted for the online intervention. Telephone interviews were conducted within 2 weeks of completion of the intervention.

### Measures

Sixteen one-to-one telephone interviews were conducted within 2 weeks of completion of the MBSH intervention. Interviews were based on the Change Interview (Elliott et al. 2001), a semi-structured interview designed to explore participants’ experiences of psychological intervention. Reflective listening techniques (Stiles 1993) were used in order to respond sensitively to experiences that emerged during the interviews. Example questions from the Change Interview included “can you sum up what has been helpful about your course so far?”; “what kinds of things about the course have been hindering, unhelpful, negative or disappointing for you?”; “what things in your current life situation have helped you make use of the course?”; and “what personal weaknesses do you think have made it harder for you to use course?”

The interviewer had no involvement in organising and conducting the intervention. There was no contact between the interviewer and the participants prior to the interviews being conducted. The interviewer was acquainted to the MBSH book but not to the online intervention. Limitations of this prior knowledge are discussed in the following. The interviews lasted between 27 and 54 min (mean 34 min). All interviews were audio-recorded and transcribed verbatim by the first author. All written transcriptions were checked against the audio recording to ascertain accuracy by the first author. Identities of all participants were removed from transcripts to ensure anonymity.

### Data Analyses

Since the research question was to identify facilitators and hindrances in engagement in MBSH, a thematic analytic method was considered suitable for the current question (Creswell 2012). Additionally, an advantage of thematic analysis is its theoretical and epistemological independence and flexibility (Braun and Clarke 2006). Thematic analysis involves a process of systematically working with the data, giving equal attention to each data item and identifying interesting aspects that form repeated patterns across the data set (Braun and Clarke 2006). Inductive coding (Boyatzis 1998) was used to code the data by the first author, followed by consultation with the third author. Inductive coding (Boyatzis 1998) is where the researcher approaches the data with a bottom-up approach, without a preconceived theoretically derived coding framework. The interviews were re-read and literature referenced, and the third author was consulted before the codes were interpreted. The coding process consisted of six phases recommended in the good practice guideline, and the researcher moved between these phases (Braun and Clarke 2006). The phases were familiarisation with transcripts, forming initial codes, searching for themes, reviewing themes, defining and naming themes and producing reports. Brief memos elaborating relationship of the codes were prepared to organise the codes in a theme. Once the initial coding was completed, the codes were examined for common patterns and dissimilarities across the codes. Transcripts were separately analysed, and emerging themes were marked. This was followed by merging or differentiation of themes that emerged into overarching themes. Although the themes may have been influenced by the primary research questions, no pre-existing theories or coding frames were used.

Three credibility and reliability checks were conducted. First, the first and third authors conducted a consensus review and appraisal of themes from each transcript. Second, two independent assessors with limited knowledge of the research question were allocated 40 sample quotations from the transcripts to allocate to a list of themes. Since a high (87%) inter-rater agreement was achieved (Joffe and Yardley 2004), no further changes were made to the themes. Third, the overarching themes were reviewed by the second author along with some sample quotations from the interviews. During each of these three stages, where there was disagreement, consensus was reached through discussion among the raters. No significant omissions were suggested.

Pseudonyms are used throughout to protect participants’ anonymity.

## Results

Four overarching themes of facilitation and hindrance to engagement were identified, namely, “attitude towards engagement”, “intervention characteristics”, “process of change” and “perceived consequences”. The themes and sub-themes are described, followed by a narrative account of the themes in Table 1. Themes emerged through engagement in the book-based and web-based mindfulness self-help interventions were analogous, and no significant dissimilarities were noted between the intervention types and so these are presented together.

**Table 1:** Overarching themes, themes and sample quotes

Overarching themes	Themes, sub-themes and sample quotes
Attitude towards engagement	*Motivation to reduce stress*: “I am always keen to learn how to manage stress better”.
	*Prior knowledge*: “I had heard about mindfulness from colleagues, so always wanted to try it out”.
	*Positive predisposition*: “I feel I was already mindful before the course started, so the practice didn’t feel strange, you know”.
Intervention characteristics	Rationale *Belief in the rationale of mindfulness*: “The justification given about how this [mindfulness] works, kept me motivated to keep carrying on [practice]”. *Lack of rationale*: “Maybe a better explanation of why this [thinking about my problems] was not helpful would help, because unless I think about my problem how can I solve it!”
	Types of practice *Length of practices*: “Some of those [practices] were so long, I used to fall asleep” “I have noticed I am able to do the brief practice like 3-min breathing even when I am in a lot of stress”. *Intensity of the intervention*: “The course was too intense for me … there was too much to do so I gave up”
Change process	Becoming more mindful *Decentering*: “I understood my mind is only a part of me … so I can take a step back and read my own mind”. *Present moment focus*: “It [sitting mindfulness meditation] helped me find and anchor to the present moment … I realised the current situation is not as stressful as I felt”.
	*Habitual perseveration*: “It was hard to stop myself from thinking about my to-do list, so I wanted to give up”.
Perceived consequences	Perceived effects of mindfulness on mental health and well-being *Improved well-being*: I have noticed I am calmer now when there is stress” *Emerging negative thoughts*: “The thoughts you want to shut down comes to you easily during meditation. I once had to stop meditating because I didn’t want to think about it, it made me sad”.
	Change in self-compassion *Increased self-compassion*: “I think it is good to practice [mindfulness] because it helps you accept your flaws and its O.K [to have flaws]”. *Becoming self-critical*: “I felt I wasn’t motivated [to practice meditation] because I was being harsh and critical of myself all the time. I felt, this is not difficult why can’t I get it”.
	*Increased sense of agency over thoughts*: My mind always kept thinking … but now I can notice [my thoughts] and respond. I feel I have more control over my mind now”.

### Attitude Towards Engagement

This overarching theme describes participants’ intentions of engagement before the intervention started. It also considers their perception of whether mindfulness is easy or difficult to engage with based on their personal dispositions. Three themes were identified under this overarching theme.

#### Motivation to Reduce Stress

Participants described their prior interest and positive attitude towards engaging in the intervention in order to manage stress better. This theme facilitated engagement as it seemed participants were already considering engaging with the intervention before it had started. For example,

“My job is so stressful; I felt I needed to learn it (mindfulness) so I could reduce my stress” (Sarah).

“So I thought (by learning mindfulness) I’ll get to learn strategies to manage stress at work and also in life” (Victoria).

#### Prior Knowledge

Participants’ comments reflected that positive feedback and promising research findings had an impact in facilitating engagement in the intervention. Awareness of the effectiveness of mindfulness appeared in almost all of the interviews and was often described as the main precursor of willingness to participate in the intervention. For example,

“I talk about mindfulness all the time at work. Often we recommend mindfulness to patients because you know, it is really effective. And also so many of my patients said that it has changed their lives. So, when I got this opportunity, I said to myself I have to try it” (Anna).

“I wanted to participate because I knew that research says it (mindfulness) is effective for people with depression. I think it should be effective for us too so I wanted to help your research” (Emilia).

These responses from participants indicate awareness, positive attitude and curiosity to understand and learn mindfulness skills and hence facilitated motivation to engage prior to and during the intervention.

#### Personal Predisposition

Some participants noted that mindfulness was similar to their natural coping style, and hence, engaging in the intervention was not perceived as an extra undertaking. This perception of predisposition was mainly with respect to mindful daily activities, for example, “It felt really natural, I love nature so noticing nature was not unusual for me” (Emilia). The observation of a personal predisposition occasionally was noted with regard to more formal mindfulness practices, for example, “I didn’t know about the 3-min breathing space before (the intervention started), but I think I have always done it, especially before important meetings. I always tend to pause and relax for a bit” (Katie).

### Intervention Characteristics

As an overarching theme, this encompasses the facilitators and barriers related to the materials provided and the practices suggested in the intervention.

#### Rationale

This theme was noted as a twofold theme, working as a facilitator for some participants and a hindrance for others. Some participants noted that a reasonable rationale was provided in the intervention. Often, it was noted as a motivation to practice, for example, “I didn’t know about this technique of thinking. It (intervention) was explained nicely so you know how this (mindfulness) works and why worrying might not always be good. That kept me going” (Amber). Some participants noted a good rationale as a facilitator that restricted disengagement, such as “it is very important for me to understand what I am doing and why, I guess if I didn’t understand the logic clearly I would have given up” (Martha).

Contrastingly, some participants noted that the rationale was not robust, thereby serving as a hindrance to engagement. Some participants noted that the purpose of mindfulness was merely to distract oneself from worries and stress, for instance, “if there would be more clarity of how mindfulness works I might have given it another go. I understand worrying might not help but how this (mindfulness) would help I am not sure” (Charlotte).

#### Types of Practice

One of the most frequently noted themes in the interviews was the facilitators and barriers associated with the type of practice in the intervention. No contradictions were noted in this theme, and the fundamental concern was apparent.

#### Length of Practices

Participants reported that the longer practices such as body scan and sitting meditation were more challenging to engage with compared to shorter practices. For example,

“To be honest, I enjoyed the overall experience and I think I have learnt mindfulness, but I can’t do another body scan, it is way too long for me” (Adam)

“I see why you need to do it (sitting meditation) but I used to find it very uncomfortable. I don’t think we are designed to sit for that long” (Rose)

Shorter practices, on the other hand, were noted as a facilitator of engagement, for example, “I am still practising the breathing exercise. I think I’ll make a habit of it” (Grace) and “I struggle to make time for things at work, but the short practices I can do during lunch” (Sophie). Shorter practices and mindful activities were also considered as a facilitator due to the ease of practice, such as “I used to walk to work and now I mindful walk to work. I think it (mindful walking) is short and easy to fit into your schedule” (Katie).

##### Intensity of the Intervention

Some participants disengaged from the self-help intervention because of the perceived demands of the course, “When I signed up for this (intervention), I had no idea there would be so many things to do every day. I wouldn’t have signed up had I known. I don’t have the time” (Chloe). Others felt that a reduction in the intensity of the course might have led to increased engagement, for instance, “I liked what I was doing to be honest. Had there been less number of things (practices), I might have continued practice” (Ivy).

### Change Process

This overarching theme describes changes brought about during mindfulness practice in the intervention. This overarching theme is twofold, facilitating as well as hindering the process of engagement in the intervention. One participant summarised the process of participation in the intervention. For example, “It (mindfulness) might help you or it might not, but I do think through these practices you get to know yourself and your surroundings better” (Jessica).

#### Becoming More Mindful

Most participants noted that some changes brought about by participating in the intervention changed their “way of being”, and this in turn motivated them to engage more. Several participants noted how they learnt to decenter, for example, “I realised we think all the time and then our thoughts become reality. You don’t realise how this affects you. I can now understand when I am overthinking and I step back for a bit. I didn’t know you could do this. The more I meditate the better I get at stepping back” (Anna) and “I realised I am always on auto-pilot. It has become a habit, you know. As the weeks went by I realised I am changing, so I kept going (practising) (Amber).

Some participants noted that the benefits of present moment focus facilitated their engagement in the intervention. For example, “The anchor thing was the most important learning for me. When I stop practising I tend to lose it, so I try to keep practising when possible” (Adam) and “I think this is a new way of living really. When I am at present, I can see things more clearly. It is difficult to get it at first but I got better with practice” (Grace).

#### Habitual Perseveration

Although the process of changing the way of being facilitated engagement for some participants, others felt that it was difficult to achieve and they had difficulty shifting from their pre-existing cognitive styles. For example, “I know it (mindfulness) is supposed to be good for you, but I am a do-er. I like to think about my problems and sort them out. I found it difficult to sit through the practices, so I gave up” (Rose). Some participants actively used the practice time for perseverative thinking for instance, “I have a busy life, I can’t stop and concentrate on my breathing, (and) I don’t have the time. To be honest, I sometimes used the meditation time to mentally make my to-do list. I realised I am not being able to do it right so I dropped it” (Sophie).

### Perceived Consequences

This overarching theme describes the participants’ perception of the impact and consequences of taking part in the intervention. These include consequences of each practice and the complete intervention. As a twofold overarching theme, there was facilitation as well as hindrance to engagement.

#### Perceived Effects of Mindfulness on Mental Health and Well-Being

Most participants described the effects that the intervention has had on them and how this had influenced that level of engagement in the intervention.

Most participants noted that practising mindfulness made them calm, for example, “My job is very stressful and I am usually quite anxious at work. My colleague pointed out the other day that I have slowed down, I am calmer now. I definitely think it is because of this course so I am not going to give it up” (Martha). Other participants pointed out how the intervention had helped them manage their emotions better, for instance, “I used to get very angry very quickly. I have noticed I don’t get angry so easily, it is probably because I am handling stress better these days, so I am planning to continue practice” (Emilia).

For some participants, however, practising mindfulness had a contrasting effect emotional well-being, for example, “I had recently had a bereavement in the family, the meditation brought all the memories back and I just couldn’t handle it. I had stopped practising from then on” (Chloe). For some participants, more general negative thoughts emerged as a result of mindfulness practice, such as “When you meditate, thoughts that you have been avoiding creep up on you, like your work stress, debts. I thought meditation would help me but it made me more nervous, so I didn’t practice as much I was supposed to” (Charlotte).

#### Change in Self-Compassion

As a theme, change in self-compassion acted as a facilitator for some and hindrance for others. Some participants noted their self-compassion increased during the intervention, helping them to continue practice, for example, “I used to get really harsh on myself, especially with work-related stuff. I never realised this before participating (in the intervention). I am more kind to myself now, so it has helped me, I should probably practice more” (Jessica).

Some participants, however, noted that mindfulness practice made them more critical, for example, “I know it is supposed to work but I don’t think it did for me. I used to get really worked up about not getting the point, I don’t know if it is just me but I was demanding more and more from myself. So finally I gave up” (Jessica). For some participants, however, not practising as opposed to not “getting mindful” led to self-criticism, for instance, “I take my to-do list very seriously but I couldn’t make time. I realised I was getting bitter because I was not practicing so I finally removed it from my list of things” (Ivy).

#### Increased Sense of Agency Over Thoughts

A very common theme that emerged from the interviews was the participants’ increased sense of agency over thoughts and how this improved engagement, for instance, “I feel more in control of myself and less regulated by my mind. It is a good feel; I feel more liberated … I think I will continue mindfulness” (Katie). One participant summarised this theme as,

“It was like learning to swim. You don’t always swim but once you know how to you will never drown. I now know about mindfulness and the being mode, I can use it when I am stressed. As long as I keep practising I will never get overstressed, which is how I see this. I have more control now” (Sarah).

## Discussion

**Fig. 1:**
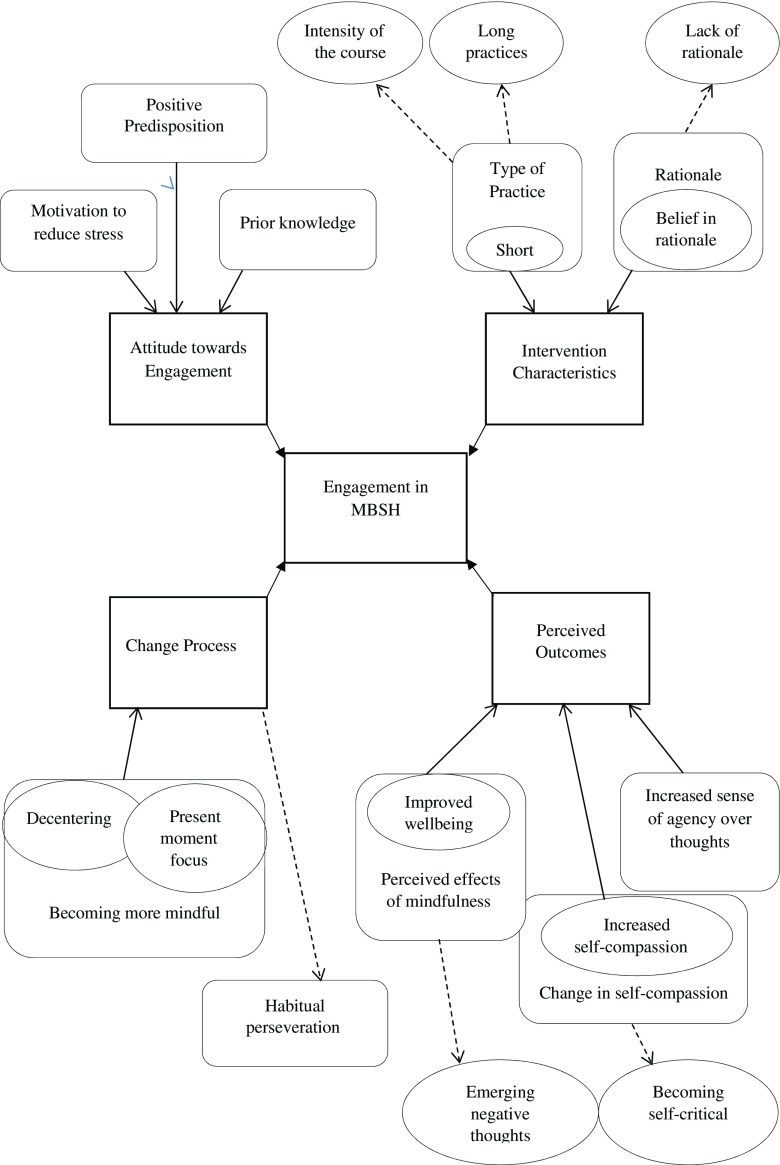
A model of the facilitators and barriers of engagement in MBSH interventions. Hindrances are marked by *dashed arrows going outwards*, whilst facilitators are *arrows going inward*

This study aimed to identify the facilitators and barriers to engaging in a MBSH intervention with healthcare staff using thematic analysis to analyse qualitative interviews (Fig. 1). The overarching themes that appeared to influence participants’ engagement were *attitude towards engagement* in the intervention such as motivation to reduce stress, prior knowledge and positive predisposition; *intervention characteristics* such as length and intensity of practices; *change processes* such as becoming more mindful and habitual perseveration; and participants’ *perception of consequences*, such as improved well-being, change in self-compassion and increased sense of agency over thoughts.

The attitude towards engagement in MBIs consisted of themes of motivation to reduce stress or the perceived need of learning mindfulness and a perception of being positively predisposed or being naturally mindful. *Prior knowledge* of the effectiveness of mindfulness techniques also emerged as a facilitator of engagement in MBIs. This is similar to previous finding of expectancy research that suggests a link between a high expectancy of change with greater compliance with homework tasks within CBT (Westra et al. 2007). It was interesting to note that the themes around attitude towards engaging in the MBSH were all facilitators, suggesting that participants that were interviewed started the intervention with an intention of engaging with it. In his well-established theory of planned behaviour, Ajzen (1985, 2012) stated that behavioural intentions can be used directly to predict behavioural achievement. Hence, a positive intention or attitude towards engagement may enhance the level of engagement. However, this might also be reflective of sampling bias as all of the participants in this study volunteered for to participate and to be interviewed following use of their chosen MBSH intervention.

The overarching theme of intervention characteristics clearly indicated the difficulty of engaging with longer practices and, in contrast, the relative ease of incorporating shorter practices in daily life. The number of practices or intensity of the intervention also hindered engagement. This is similar to previous research that indicated that conflicting demands and time are one of the key hindrances to engaging in MBIs more broadly (Moore and Martin 2015). This hindrance may also be typical of the current sample group as the workload in NHS is often reported as heavier than other professions (Weinberg and Creed 2000), and hence, the long practices may be particularly difficult to fit into the schedules. MBIs for healthcare staff could be tailored to incorporate shorter practices aimed at bringing mindful awareness to daily life activities. However, further research is needed to explore the effectiveness of shorter mindfulness practices as formal mindfulness meditation exercises (such as body scan and sitting meditation) are more often reported to be associated with improvement in most facets of mindfulness (Carmody and Baer 2008; Hawley et al. 2014).

*Decentering and focussing on the present moment* were indicated as facilitators of engagement in MBIs. This indicates that participants who perceived themselves as *becoming more mindful* remained more engaged with the intervention. This is comparable to previous findings that suggest that a main struggle in engaging in guided mindfulness interventions for some participants is the difficulty “grasping the core concepts of mindfulness” (Wyatt et al. 2014, p. 223) and uncertainty about if they have “got the idea” (Moss et al. 2008, p. 137) of mindfulness. During the practices, getting caught up with habitual thinking was identified as one of the hindrances of engagement. This is predictable as perseverative thinking styles, such as rumination and worry, are antagonistic to the decentering processes involved in mindfulness (Wells 2005). Moreover, previous studies suggest that participants of MBIs struggle to engage primarily due to resistance to altering habitual thinking styles. A grounded theory study on mindfulness practice reported that one of the main reason participants disengaged was the urge to “do” rather than to “be” (Langdon et al. 2011). This might be especially true for the current sample as their demanding work life might reinforce the habit of *doing* over *being*. Moreover, some participants in this study noted that they “used the meditation time to mentally make ... to-do lists”. This is an important issue as this might suggest that participating in the intervention can in fact activate perseverative thinking, such as rumination and worry, that is known to implicate in the maintenance of, respectively, depression and generalised anxiety disorder (Kertz et al. 2015). It is particularly important for MBSH as the participants have limited or no support from a trained mindfulness teacher, who might encourage reconnection with the intention of mental activities during meditation practice (e.g. coming back to the breath).

*Perceived consequences* are paramount to continuing engagement with any intervention. Predictably, positive perceived consequences of MBSH enabled engagement whilst negative perceived consequences obstructed engagement. The key facilitator for participants’ engagement was the perception of improved psychological well-being. This relates strongly to previous research suggesting improvements in psychological well-being and association between regular practice (Finucane and Mercer 2006). One of the significant benefits of MBIs is an increase in self-compassion (Birnie et al. 2010). Themes from the current study demonstrate that perceived increase in self-compassion also facilitates engagement. Increase in self-compassion and improved relationship to self and others have been reported as an important theme of participating in guided mindfulness interventions in previous research (Wyatt et al. 2014). One of the commonly emerging themes of participating in guided mindfulness interventions is an increased sense of agency over thoughts (Wyatt et al. 2014). This positive consequence translated to increased engagement in the intervention. Although most participants described this theme as having “more control” over thoughts, further elaboration revealed that it was the increased awareness of thoughts and thought patterns that enhanced acceptance and increased their perception of “control”. Some participants struggled to engage with mindfulness due to the perceived negative consequences such as difficulty tolerating negative thoughts that emerged as a result of mindfulness practice and becoming self-critical due to guilt of disengaging from practice. Emerging negative thoughts during mindfulness practice has been previously noted as a key struggle to engagement. For example, Finucane and Mercer (2006, p. 7) reported that practicing mindfulness meditation led some participants to become more distressed; for example, one participant with history of childhood abuse became aware of “horrible feelings through the body” that he/she “had never felt before”. This may be difficult to tolerate working alone and suggests that for at least some people, support from a trained mindfulness teacher may be essential in order to tolerate such memories and the feelings associated with their experience. MBSH interventions may aim to incorporate psychoeducation or virtual support in order to address this hindrance. Finally, self-criticism and guilt due to slipping out of the practice cycle were also reported in previous research (Langdon et al. 2011). However, paradoxically, mindfulness practice is reported to reduce self-criticism (Birnie et al. 2010). This might emphasise the need of having some form of trained support during participating in an MBI as self-criticism is known to predict poorer treatment outcome for mental health problems such as depression (Marshall et al. 2008).

The support of group and therapist has always emerged as a crucial theme in the experience of participating in MBIs (Wyatt et al. 2014). Interestingly, the lack of trained support or support from group members was not identified as a hindrance by any participants in this research. This is encouraging since self-help-based MBIs have been found to be effective (Cavanagh et al. 2014; Spijkerman et al. 2016) and are easier to deliver. However, this should be interpreted cautiously as the participants in the current study consented to participate in an MBSH and may have been more positively predisposed to this kind of self-guided learning process. Moreover, the participants might not have discussed the lack of support or group in the interviews as they volunteered for a self-help intervention or might not be aware of the additional support that is absent from these self-help interventions. The lack of support, however, may have translated to other hindrances such as difficulty dealing with negative thoughts and feelings and not being able to break the cycle of habitual perseveration. Future research can compare the current findings with themes emerging from partially supported MBIs.

### Strengths and Limitations

The primary limitation of this study was that only 16 of the 31 study participants were interviewed. Although this is not unusual for qualitative studies and collecting data from large sample is not crucial for qualitative analyses (Marks and Yardley 2004), the data obtained from these participants may not be representative of all the participants who took part in the wider study; e.g. the participants interviewed may have more positive views and experiences of engaging in the intervention than the participants who decided not to be interviewed. Moreover, participants who dropped out may have provided novel themes on the experience of participating in MBIs that led them to drop out from the intervention. Second, the NHS staff interviewed in the current study were all from the same region, working in a mental health trust and presumably had a positive attitude towards engagement (as they had self-selected to take part in the study), and hence, the results might not be widely generalisable. Third, whilst an inductive coding approach was applied when analysing the data, we acknowledge that a pure inductive approach, whereby the researchers’ prior knowledge and assumptions do not influence coding, may not be realistic. In relation to this issue of reflexivity (Elliott et al. 1999; Yardley 2000), all authors are involved in researching self-help-based MBIs and all authors have completed an MBCT course as a participant, whilst the third author is also an MBCT teacher. It is likely therefore despite taking an inductive approach to coding and to the analysis that researchers’ prior beliefs, attitudes and assumptions about MBIs and MBSH would have played some role in determining the final results. It may be more accurate in future research therefore to apply an abductive coding approach (Reichertz 2007), whereby a reciprocal relationship between the data and the researcher is foregrounded. However, several steps of validation were taken in the current study in order to reduce bias and participants were made aware of the interviewer’s independence to the main study, which may have contributed to reducing bias in the participants’ responses in the interviews. Fourth, there are a wide range of MBSH resources available which differ substantially in their format and content, raising questions about the generalisability of findings from the current study to MBSH resources more broadly. The MBSH evaluated in the current study was the self-help book Mindfulness: a practical guide to finding peace in a frantic world (Williams and Penman 2011). This was chosen deliberately as it closely adheres in format and content to the MBCT curriculum, arguable the MBI with the strongest evidence for effectiveness in its group-based format (e.g. Kuyken et al. 2016), and was written by one of the pioneers of MBCT. This book was evaluated as a standalone intervention in the current study, without any additional support or guidance provided. Our findings therefore pertain to this particular book and cannot be assumed to generalise to MBSH more broadly, and we would suggest that attention is paid to the format and content of MBSH in future research as we cannot assume that all forms of self-help mindfulness will be experienced in the same ways. Fifth, respondent validation or “member checking” was not built into the current analysis. Future research should ensure that this important practice is undertaken in order to validate the researchers’ understanding of the participants’ subjective experiences. Whilst this study has some areas for possible improvement, it is useful in highlighting the facilitators and barriers of engaging in pure self-help-based mindfulness interventions.

Further research can explore the possibilities of specifically tailoring MBIs for healthcare professionals, which might include briefer practices (and exploring the impact of this on outcome) and greater emphasis on incorporating mindfulness in the workplace, including when working with patients. In addition, given the experiences of some participants in this study, future research could also explore the potential benefits of providing mindfulness teacher support for MBSH (e.g. by telephone or online).
